# Enabling equitable and affordable access to novel therapeutics for pandemic preparedness and response via creative intellectual property agreements

**DOI:** 10.12688/wellcomeopenres.22645.1

**Published:** 2024-07-15

**Authors:** Ed J. Griffen, Pascale Boulet, ASAP Discovery Center, Randy Albrecht, Ali Akbar, Isabella Amick, Jasmin Cara Aschenbrenner, Blake H. Balcomb, Haim Barr, Harish Battu, Jared Benjamin, Jesse Bloom, Melissa Boby, Matt Bogyo, Daniel Bolon, Pascale Boulet, Ariana Brenner Clerkin, Richard Cadagan, Eda Capkin, Maria Castellanos, Anu V Chandran, Shiyu Chen, John Chodera, Daniel Cole, David Cousins, Sara Cuadrado, Andre Schutzer de Godoy, Adeeba Dhalech, Randy Diaz, Maria DiPoto, Jennifer Doherty, David Dotson, Sarah Duggan, Matthew Evans, Michael Fairhead, Gwendolyn Fate, Daren Fearon, Oleg Federov, Matteo Ferla, Lucas Ferreira, Kate Fieseler, Mihajlo Filep, Daniel Flores, Julia Flynn, Laurent Fraisse, Gabizon Ronen, Barien Gad, Adolfo Garcia-Sastre, Christine Gathecha, James Gayvert, Charline Giroud, Carlos Godoy Parejo, Erica Goldberger, Mathew Golding, Mary Elizabeth Gordon, Ed Griffen, William Hannon, Haithem Hassan, Mike Henry, Rebecca Hertzman, Joshua Horton, Feiyang Huang, Qiu Yu Huang, Christopher Iacovella, Farren Isaacs, Dan James, Maggie Jameson, Benjamin Kaminow, Ian Kenney, Nate Kenton, Karla Kirkegaard, Lizbe Koekemoer, Nese Kurtyilmaz, Van La, Noa Lahav, Megan Lambert, Maayan Lavie, Alpha Lee, Bruce Lefker, Kendall Lemons, Brett Lindenbach, Ryan Lithgo, Ella Livnah, Nir London, Nick Lynch, Hugo MacDermott, Elizabeth MacLean, Peter Marples, Brian Marsden, Briana McGovern, David Minh, Michelle Mugeni, Almog Nadir, Nguyen Luong, Xiaomin Ni, Sam Nitz, Hnin Ookhin, Alexey Orlov, Alex Payne, Rebecca Pearl, Nichole Pedowitz, Siyang Peng, Ben Perry, Iván Pulido, Dattatraya Rawal, Lauren Reid, Michael Retchin, Ryan Richardson, Emily Ripka, Matt Robinson, Ralph Robinson, Mario Rodriguez, Grace Roper, Romel Rosales, Madihah Salim, Jenke Scheen, Celia Schiffer, Yoav Shamir, Ala Shaqra, Cole Shaw, Oded Shertzer, Sukrit Singh, Peter Sjo, Jessie Slater, Ashley Souffront, Jessica Stacey, Harriet Stanway - Gordon, Tyler Starr, Hannah Stewart, Tamas Szommer, Windy Tanner, Veena Thomas, Warren Thompson, Jason Tierney, Barr Tivon, Charlie Tomlinson, Thomas Vargo, Fernando Villalo, Annette von Delft, Frank von Delft, Martin Walsh, Wang Sije, SiYi Wang, Yuanqing Wang, Michael Waring, Kris White, Conor Wild, Craig Wilen, Eleanor Williams, Michael Williams, Max Winokan, Nathan Wright, Mary-Ann Xavier, Jerry Yap, Ivy Zhang, Jiyun Zhu, Thomas Zwaka, COVID Moonshot, Matthew C. Robinson, Nir London, Efrat Resnick, Daniel Zaidmann, Paul Gehrtz, Rambabu N. Reddi, Ronen Gabizon, Haim Barr, Shirly Duberstein, Hadeer Zidane, Khriesto Shurrush, Galit Cohen, Leonardo J. Solmesky, Alpha Lee, Andrew Jajack, Milan Cvitkovic, Jin Pan, Ruby Pai, Emily Grace Ripka, Luong Nguyen, Mikhail Shafeev, Tatiana Matviiuk, Oleg Michurin, Eugene Chernyshenko, Vitaliy A. Bilenko, Serhii O. Kinakh, Ivan G. Logvinenko, Kostiantyn P. Melnykov, Victor D. Huliak, Igor S. Tsurupa, Marian Gorichko, Aarif Shaikh, Jakir Pinjari, Vishwanath Swamy, Maneesh Pingle, Sarma BVNBS, Anthony Aimon, Frank von Delft, Daren Fearon, Charles W.E. Tomlinson, Blake H. Balcomb, Ryan M. Lithgo, Jasmin C. Aschenbrenner, Louise E. Dunnett, Alice Douangamath, Alex Dias, Ailsa Powell, Jose Brandao Neto, Rachael Skyner, Warren Thompson, Tyler Gorrie-Stone, Martin A. Walsh, C. David Owen, Petra Lukacik, Claire Strain-Damerell, Halina Mikolajek, Sam Horrell, Peter G. Marples, Lizbé Koekemoer, Tobias Krojer, Michael Fairhead, Elizabeth M. MacLean, Andrew Thompson, Conor Francis Wild, Mihaela D. Smilova, Nathan Wright, Annette von Delft, Carina Gileadi, Victor L. Rangel, Christopher J. Schofield, Eidarus Salah, Tika R. Malla, Anthony Tumber, Tobias John, Ioannis Vakonakis, Anastassia L. Kantsadi, Nicole Zitzmann, Juliane Brun, J. L. Kiappes, Michelle Hill, Karolina D Witt, Dominic S Alonzi, Laetitia L Makower, Finny S. Varghese, Gijs J. Overheul, Pascal Miesen, Ronald P. van Rij, Jitske Jansen, Bart Smeets, Susana Tomásio, Charlie Weatherall, Mariana Vaschetto, Hannah Bruce Macdonald, John D. Chodera, Dominic A. Rufa, Matthew Wittmann, Melissa L. Boby, Michael Henry, William G. Glass, Peter Eastman, Joseph E. Coffland, David L. Dotson, Ed J. Griffen, Willam McCorkindale, Aaron Morris, Robert Glen, Jason C. Cole, Richard Foster, Holly Foster, Mark Calmiano, Rachael E. Tennant, Jag Heer, Jiye Shi, Eric Jnoff, Matthew F.D. Hurley, Bruce A. Lefker, Ralph P. Robinson, Charline Giroud, James Bennett, Oleg Fedorov, St Patrick Reid, Melody Jane Morwitzer, Lisa Cox, Garrett M. Morris, Matteo Ferla, Demetri Moustakas, Tim Dudgeon, Vladimír Pšenák, Boris Kovar, Vincent Voelz, Anna Carbery, Alessandro Contini, Austin Clyde, Amir Ben-Shmuel, Assa Sittner, Boaz Politi, Einat B. Vitner, Elad Bar David, Hadas Tamir, Hagit Achdout, Haim Levy, Itai Glinert, Nir Paran, Noam Erez, Reut Puni, Sharon Melamed, Shay Weiss, Tomer Israely, Yfat Yahalom-Ronen, Adam Smalley, Vladas Oleinikovas, John Spencer, Peter W. Kenny, Walter Ward, Emma Cattermole, Lori Ferrins, Charles J. Eyermann, Bruce F. Milne, Andre S. Godoy, Gabriela D. Noske, Glaucius Oliva, Rafaela S. Fernandes, Aline M. Nakamura, Victor O. Gawriljuk, Kris M. White, Briana L. McGovern, Romel Rosales, Adolfo García-Sastre, Daniel W. Carney, Edcon Chang, Kumar Singh Saikatendu, Laura Vangeel, Johan Neyts, Kim Donckers, Dirk Jochmans, Steven De Jonghe, Gregory R. Bowman, Bruce Borden, Sukrit Singh, Andrea Volkamer, Jaime Rodriguez-Guerra, Gwen Fate, Storm Hassell Hart, Kadi Saar, Benjamin Perry, Laurent Fraisse, Peter Sjö, Pascale Boulet, Sophie Laghnimi-Hahn, Charles E. Mowbray, Lauren Reid, Paul Rees, Qiu Yu Judy Huang, Sarah N Zvornicanin, Ala M. Shaqra, Nese Kurt Yilmaz, Celia A. Schiffer, Ivy Zhang, Iván Pulido, Jenny C. Taylor, Tristan Ian Croll, Lennart Brewitz, Lulu Kang, Van Ngoc Thuy La, Amna Haneef, David Minh, Mihajlo Filep, Jenke Scheen, Benjamin Kaminow, Robert Arbon, Alexander Matthew Payne, David Schaller

**Affiliations:** 1 ASAP Discovery Consortium and MedChemica Consultancy Ltd, Macclesfield, Cheshire, SK11 6DU, UK; 2 Drugs for Neglected Diseases initiative (DNDi), Geneva, 1202, Switzerland

**Keywords:** Pandemic Preparedness, novel therapeutics, intellectual property, equitable access, patents, licensing

## Abstract

The COVID-19 pandemic demonstrated that the current purely market-driven approaches to drug discovery and development alone are insufficient to drive equitable access to new therapies either in preparation for, or in response to, pandemics. A new global framework driven by equity is under negotiation at the World Health Organization to support pandemic preparedness and response. Some believe that the global intellectual property (IP) system itself is part of the problem and propose a purely Open Science approach. In this article, we discuss how existing IP frameworks and contractual agreements may be used to create rights and obligations to generate a more effective global response in future, drawing on experience gained in the COVID Moonshot program, a purely Open Science collaboration, and the ASAP AViDD drug discovery consortium, which uses a hybrid, phased model of Open Science, patent filing and contractual agreements. We conclude that ‘straight to generic’ drug discovery is appropriate in some domains, and that targeted patent protection, coupled with open licensing, can offer a route to generating affordable and equitable access for therapy areas where market forces have failed. The Extended Data contains a copy of our model IP policy, which can be used as a template by other discovery efforts seeking to ensure their drug candidates can be developed for globally equitable and affordable access.

## Plain language summary

Drug discovery and development organizations usually recoup their investment in this risky and expensive process by filing patents on drug candidates which, if granted, give them a time-limited monopoly on the manufacture, sale or licensing of the drug. This means they can negotiate its price and terms of distribution, which creates distortions in access globally. In an alternative ‘Open Science’ approach, R&D organizations publish all the information about a prospective drug without applying for patents, meaning that anyone can use this knowledge to make and sell the drug, while the R&D organizations have no control over how it is priced or distributed.

In a pandemic, fast-spreading viruses must be rapidly contained by delivering drugs to where they are most needed. This requires innovation and global access, but this is stifled in both models – in the first because of patent abuses, in the second because the lack of control may jeopardize the most efficient development.

The authors share a model that prioritizes globally fair and affordable pricing by creating ‘maximally permissive licenses’ based on ‘minimally defensive patents’. They explain the practical and bioethical background to their proposals and share an example of collective management of intellectual property and licensing agreement that is being used in the AI-driven Structure-enabled Antiviral Platform (ASAP) Center’s Pandemic Preparedness work.

## Introduction

**Nobody is safe unless everybody is safe.** Modern air travel means that infectious agents can spread easily across the globe. A local outbreak that begins in a high-density population area can rapidly become a global problem ^1^. Antiviral therapeutics represent one critical component in the drive to preventing future pandemics ^2^. As the climate crisis pushes various insect species to higher latitudes, diseases currently only spreading in low and middle income countries (LMICs), such as Dengue and Zika, are likely to migrate to high income countries (HICs). If we are to contain future epidemics before they become global pandemics, open collaboration frameworks will be needed to generate new anti-infective therapies that can be rapidly made available globally in an equitable and affordable manner. While a new global framework is under negotiation at the World Health Organization to support pandemic preparedness and response ^3^ we believe there are opportunities than can be taken immediately. Over the last three years we have worked in an international consortium on a response to the COVID-19 pandemic (the COVID Moonshot Consortium) ^4^ and now are generating new agents in preparation for future pandemics (via the ASAP Discovery Consortium). However, this has not been without its challenges. One of the non-scientific challenges has been how to negotiate intellectual property (IP) agreements that ensure the discovery, development and equitable distribution of such therapies at affordable prices. This article will explore the issues involved and share our learnings from the Moonshot Consortium and the framework that we are using in the ASAP AViDD drug discovery consortium ^5^.

## Drug discovery, development and intellectual property

Drug discovery, clinical development, manufacturing and distribution are highly complex interdisciplinary scientific, technical, regulatory and logistic endeavors requiring significant investment ^6^, with a lot of variation depending on the field of research, existing therapies and the origin of the investments. It is also highly risky with high attrition rates in both discovery and development programs. Most lucrative drug discovery research takes place in the commercial sector, with the investment secured by patent protection of novel pharmaceuticals. These patents are then typically sold or licensed to pay for the investment in research and development and to generate a return, however, this often happens without equitable access in many countries. The most important patent is on the novel chemical structure of the therapeutic agent, known as a ‘composition of matter’ patent. In exchange for full disclosure of the chemical structure and the manner of making it at the time of filing, the patent owner is granted a time-limited, exclusive right to prevent others from exploiting the compound for the use described in the patent without the owners’ consent. The original aim of the patent system is to create a global benefit for society by requiring full disclosure of the method for reproducing the patented invention and, once the period of exclusivity is ended, the invention may be reproduced by anyone without the need for a license. There are however criticisms that full disclosure of the best mode to carry out the patented invention is rarely made. The alternative is to protect therapeutic agents as ‘Trade Secrets” (instead of patents), i.e., not disclosing them, meaning that so long as the secret is maintained, the owner of the invention maintains a monopoly. This is feasible in some areas of technology, however pharmaceuticals are, by their nature, extremely tightly regulated in terms of their purity and, therefore, it is easier to determine their nature, which makes maintaining the composition of a pharmaceutical secret technically unlikely. Using Trade Secrets also drives a culture of secrecy and precludes further innovations in an area where having multiple therapies available is a defense against multiple threats from the same viral family as in SARS-CoV-1, MERS and SARS-CoV-2 as well as emergence of resistance in one virus as is observed in HIV.’

In the pharmaceutical sector, this life cycle of innovation, return and transition to generic medicines is reflected in the behavior of the market, with both large pharmaceutical companies and venture capitalists making the large early stage investments, often with financial support from governments, and then charging high prices to recoup their investment with a profit during the monopoly phase; prices then reduce once patent protection expires as the patent holder can no longer enforce their monopoly and the medicines can be produced competitively by generic manufacturers. This market structure has two shortcomings. First, it can impede follow-on innovation if published knowledge is not licensed, which open science approaches aim to address. Second, it creates inequities because of various scenarios: the high price of new therapies usually makes them unaffordable in many territories, diseases only prevalent in LMICs with a low return on investment are only supported by governmental and philanthropic research (‘neglected diseases’) and diseases with a small patient population (‘rare diseases’) may only receive academic or charitable research, even in HICs. In rare diseases, the combination of a small market size and a patent monopoly often leads to predatory pricing ^7^. New antibiotics to treat antimicrobial resistance (AMR) driven infections are a therapeutic class with particular problems. New antibiotics need to be used judiciously to slow the rate of resistance emergence, courses of treatment are usually short, and there is an expectation that therapies will be cheap; this results both in low volumes of sales and low prices. Therefore, despite the life-saving nature of such treatments, the estimated commercial return for new antibiotics is low and investment is sparse.

The market failure of research for new antibiotics to treat AMR driven infections ^8^, despite the global increasing threat, is a clear example of how the interaction between the legal framework for pharmaceutical research and its funding model fails to address significant patient and public health needs. Infectious diseases of pandemic potential are an even more serious challenge to the conventional pharmaceutical research and development model, since there are no, or very few, cases of the disease currently and the ideal use case would be rapid distribution and treatment to ‘blanket an area’ when an outbreak occurs, to prevent deaths in critical health workers and the global spread of the novel pathogen. A successful therapeutic agent might, therefore, remain unused for many years, be rapidly used and then not needed again. This does not fit with current conventional incentive models based on patents, as the patent might expire before any return is generated for the investor. The general lack of investment in diseases of pandemic threat prior to the COVID-19 pandemic is evidence that this model is not effective.

## Learning from the COVID Moonshot: to patent or not to patent?

During the project, we adopted a patent-free, open science, direct-to-generic approach. This was highly effective in the research phase, enabling rapid recruitment of collaborators and remarkably fast transfer of ideas and data unencumbered by lengthy contract negotiations. However, there were unforeseen consequences.

First, discussions with potential manufacturers were more complicated, because we could not guarantee that other parties would not develop production routes to the new antiviral in parallel, risking the recovery of their investment costs; this dissuaded some manufacturers from pursuing production of the antiviral. While several manufacturers have expressed interest in downstream collaboration based on anticipated Phase 2(a) clinical efficacy results and the state of the COVID-19 pandemic, investing in the project before that point would have been too great a risk.

Second, in order to provide reassurance to downstream development partners, a counterintuitive outcome was that we could not publicly disclose the chemical structure of the antiviral we chose to take into the clinic until clinical trials had been initiated. This outcome was not ideal in terms of enabling the scientific community to make progress based on our work, but was required to prevent others from performing poorly controlled experiments or filing for regulatory approval using our data in a manner that could significantly delay or impede our global access objectives. Thus, counter to the open ideals of the project, we were obliged to delay disclosure of critical data to safeguard our ability to negotiate access conditions with a development partner.

Third, several philanthropic funders were uncomfortable with the patent-free approach and declined to provide funding for the research phase of the project, because there was no patent to control downstream licensing. This delayed identification of a sponsor to finance our progress to a candidate drug. In the end, the international program Access to COVID Tools Accelerator (ACT-A) through Wellcome funded the expensive preclinical development phase of COVID Moonshot antivirals, despite the lack of a patent, with the aim to reach Phase I clinical safety readiness. 

Fourth, we underestimated the impact that our adoption of open science would have on follow-on research. In addition to numerous academic efforts around the world working with the data we released, a major pharmaceutical company built on our work to develop a new, effective antiviral that was approved in Japan and fast-tracked by the FDA ^9^, providing an important new tool for treating COVID-19.

With these lessons in mind, the ASAP consortium has refined its strategy to secure control of the antivirals it develops to make them globally, equitably, and affordably accessible, while making our data openly available as rapidly as possible.

## Making the patent system work toward global equitable and affordable access

We next explored how the patent system could be used, as a tool, to enable our goals of both equitable global access and rapid development. It is important to understand key features of patents on novel chemical matter – chemical compounds that might be useful antiviral drugs. In commercial drug discovery, patents are generally as broad as possible, with the goal of excluding competitors from making similar or second-generation molecules, even if they might be better, cheaper, or have fewer side effects. This is the opposite of what is needed in a pandemic, where second-generation antivirals that are more convenient to administer, cheaper, and/or have different resistance profiles are required, as they may be essential for saving lives.

We, therefore, propose the concept of a ‘minimally defensive patent’: a precise, focused patent, that only specifies the exact molecules to be studied clinically. This would enable us to prevent others from working on only the exact molecules we aim to take all the way through the clinic to manufacture, avoiding the issues of either ‘rogue synthesis for profit’ or uncontrolled experimentation leading to clinical development delays, while still allowing others to work on better, cheaper, or differentiated antivirals that build on our work. Further, legal advice received at the time highlighted that given variation in patent laws, it would not be possible to guarantee that a third party could not obtain patent rights that would block us, building on all our published data in the public domain. Lastly patents would also provide ASAP with solid rights to negotiate and agree the best conditions with development partners to ensure global, equitable and affordable access during clinical development, manufacture, distribution and commercialization.

As an open science program, we are still putting a wealth of data into the public domain immediately – antiviral targets, structures, protocols, plasmids, mutational data, molecules and data up to early lead compounds. During the final stage of drug discovery – lead optimization – our unsuccessful early discoveries (chemical structures and measured data) are rapidly disclosed into the public domain, while the release of the remaining data on the lead compounds is delayed. This provides contemporaneous researchers with an indication of areas that we have found to be unproductive, thus saving time and avoiding duplication of work. In addition, we aim to request that ASAP patents on the lead compounds are published at the earliest possible date after filing, rather than after the standard 18 months, so that we will be able to share all of our remaining data upon patent publication, thus avoiding the need to conceal the identity of the antivirals we are taking into the clinic. All our data will eventually be made openly available, allowing others to exploit it for further research, although any commercial exploitation of ASAP molecules will require ASAP approval.

Our policy of patenting only the compounds we plan to take into the clinic will support rapid development of our own molecules, and immediate publication of compounds that are not viewed as having potential will support others building on our work to discover differentiated antivirals that serve the public good. The combination of these two approaches will maximize our ability to deliver on our primary goal of generating new antivirals for pandemic use that will be available globally, affordably and equitably, while being as open as possible with the results of our research as quickly as possible. Beyond just publication of data, we support adopting FAIR principles ^10^, ensuring the data is distributed and then stored in multiple locations, as for future exploitation it is vital the knowledge we gain is not locked in ’data tombs’ where future drug hunters cannot readily exploit it. For the physical material, the ideal solution would also be to have samples of antivirals available globally at key centers to enable rapid testing against emergent viral threats. This means we can immediately identify therapeutic opportunities at the earliest opportunity. Then, with the combination of the historically determined ADME and toxicological profile, the profile against the new viral threat, and an instantly available licensing and IP framework, the antivirals could be put to work to contain an epidemic without further delay.

## The ASAP licensing strategy focuses on ensuring global benefit

Owning a patent for the compound we aim to take into the clinic allows us to negotiate access terms and conditions under which it will be licensed to downstream development partners, who are essential in the conducting of clinical trials and manufacturing our future antivirals. Under the ASAP IP policy, all ASAP members agree to only license ASAP patent(s) on terms that ensure equitable, global and affordable access. ASAP undertakes to license its patent rights under global non-exclusive licenses, as the preferred licensing strategy, to ensure affordable competitive pricing and sufficient global supply, if needed through multiple manufacturers. By controlling the licensing of patent rights, ASAP can provide clinical developers with the assurance that the development and commercialization of the end product will be managed to ensure both a fair return on private investment and global equitable and affordable access. Building in licensing terms that avoid the generation of monopolies is a key tactic in ensuring affordable antiviral production. The assumption is that significant public funding will be made available by the global donor community to incentivize and support the downstream development of new antivirals for pandemic use, which must be affordable and equitably available to serve the common public interest. Therefore, exclusivity on commercialization of the compound itself should not be necessary to recoup investments in downstream development. Developers would still be able to claim some IP on specific formulations or manufacturing processes that distinguish their products from competitors, but our licensing strategy would allow free access to the molecule itself for interested parties. This mirrors current practice where generic pharmaceutical manufacturers need to demonstrate bioequivalence to gain registration of their new product but may attempt to generate beneficial intellectual property through innovation. The policy also foresees that licensing of new IP generated in the downstream development of ASAP antivirals could be subject to the payment of reasonable and tiered royalties (based on countries’ gross national income) to the developer, but only for sales in HICs and upper-middle income countries.

In case of demonstrated inability to engage with a development partner on global non-exclusive licensing terms, ASAP IP policy enables ASAP institutions to decide, by vote, to license ASAP patent(s) on an exclusive basis to a development partner. However, such exclusive rights would be limited to HICs only and subject to additional conditions: first, non-exclusive licenses would remain available to interested companies for development and/or sale in LMICs, to secure the lowest sustainable prices based on generic competition. Second, the exclusive licensee would be obligated to sell ASAP antivirals at transparent and affordable prices in HICs, and to reserve half of the production for sale to LMICs on a no-profit-no-loss basis, allocated equitably according to WHO guidelines; this is to avoid sales being prioritized in HICs, as happened with first COVID-19 vaccines that were commercialized. Lastly, in the event of the World Health Organization declaring an international pandemic, ASAP IP policy requires any developer of ASAP antivirals to sub-license its rights to interested generic manufacturers, including through international patent pools, to ensure sufficient global supply.

## Could pandemic preparedness be delivered by a corporate-government response?

A critical response to the discussion above is to reflect on the success of large Pharma in rapidly bringing antiviral small molecule therapeutics to market. The first new SARS-CoV-2 inhibitor to market, nirmatrelvir, was discovered and developed incredibly rapidly by Pfizer from a SARS-CoV-1 inhibitor lead in only 21 months. Shionogi invented a second structurally completely different SAR-CoV2 inhibitor in less than 16 months ^11^. Modern large Pharma have unparalleled resources which can be applied to deliver treatments to patients faster than ever before. However, this was in both cases pandemic response not preparation. Neither company had compounds that had been put into Phase 1 trials and were ‘Phase 2 trial ready’ at the start of the COVID-19 pandemic. As a thought experiment, we could consider whether governments or philanthropic organizations might fund Pharma to conduct pandemic preparation research and under what circumstances that might be successful. The issue is not a technical one but a challenge to find a viable business case. Pharma are responsible to their shareholders, and therefore work done in delivering Phase 2 ready agents with no existing market would still have to deliver a return on investment. This would entail the funder paying a premium price to the corporate entity to carry out research and license the pandemic response therapy to ‘sit on the shelf’. Financially this is a feasible solution, however, politically, governments or NGOs may find it a challenge to persuade their funders or voters that providing significant profits to large Pharma to provide “pandemic preparedness services”, is a good use of funds unless such premium public funding would be subject to global equitable access conditions, building upon the approach chosen by ASAP. This analysis is not meant to imply that this form of public-private collaboration is impossible, but merely to point out the challenges that it holds. The private sector has shown itself to be able to respond rapidly, but has not demonstrated that it can be prepared for pandemic threats with market incentives alone, nor that it is able to ensure affordable and equitable access globally.

## Bioethical foundations

The entire preceding discussion makes certain assumptions about an underlying ethical framework. Here we clarify that all that is required is a minimal view of what is justified to reduce the risk of future pandemics. We view creating and sharing the knowledge of anti-viral medications as being a privileged class of ‘common interest objects’ ^12^. Pandemic viruses are a threat that does not discriminate between people and, therefore, is an unambiguous example of a common threat. The ‘common interest’ in this context is that one group having knowledge of how to counter the threat does not preclude another group from having it. Access to actual physical anti-viral medications themselves, however, is more complex, because although knowledge may be copied and propagated, exclusive rights on such knowledge can limit and constrain follow-on research and, if there is a limited supply of medication, this may lead to rivalry. We consider that the failure of pure free-market solutions to supply research and production of medications for pandemic preparedness justifies discovery of antivirals through common endeavor. However, when it comes to the supply and distribution of anti-virals, the nature of epidemic to pandemic biology means that there is a tension between the common and individual interest. An individual or community that hoarded an anti-viral medication to save itself from a future threat might prevent the containment of an epidemic and trigger a pandemic, perversely leading to a worse outcome for itself. This is the ultimate driver for proposing the license structure we suggest, where, as a minimum, anti-viral medications discovered by common endeavor should be distributed for common benefit. This requirement in turn justifies the protection of anti-viral drugs with limited patents to enable and protect the licensing of rights we propose.

Our proposals have a conceptual foundation grounded in extensive historical traditions in both secular and faith philosophical traditions in many cultures, where ‘taking efficient measures for public health and safety’ are recognized as a practical case where the maximum benefit for the community is gained by co-operation. There is an analogy to the Aristotelian concept of a well-ordered society generating ‘shared weapons and fortifications’ against a common enemy ^13^. COVID-19 has again demonstrated that a pandemic virus is a common threat even to the most technologically advanced communities of 21 ^st^ century humanity and thus justifies a common interest defense. These issues are discussed in a broader and more general sense in a recent WHO publication ^14^.

## Conclusion

ASAP IP policy aims to use patent rights as a tool to avoid the short-comings of a purely open-science approach, while contractually conditioning the use of patent rights to achieve ASAP’s fundamental goal of global, equitable and affordable access. We believe that the approach described can enable, enhance and accelerate the discovery of pandemic preparedness therapeutics using methods familiar to those working in the fields of drug discovery and development, and can be implemented by international donors and foundations.

This publication, along with the appended model policy (see Extended data ^15^), is part of our open innovation strategy and we hope that others will examine, adopt and improve upon our approaches.

Equitable and affordable access to therapies for potential future pandemics is neither an idealistic fantasy nor a luxury, it is a necessity for pandemic prevention. In order to ensure that the next pandemic is not even worse than those previously experienced, we need to be able to deliver effective anti-infectives to sites of outbreak as rapidly as possible, without being hampered by delays caused by negotiations that could have been conducted in advance. Continuing to do what we have done in the past will result in the same problematic outcomes in the future; as well as innovation in technology we also need innovation in funding policy, such as making public funding contingent on legal and contractual structures that favor access, as described in this article.

## Disclaimer

The views expressed in this article are those of the author(s). Publication in Wellcome Open Research does not imply endorsement by Wellcome.

The ASAP Discovery Consortium is a NIAID-funded Antiviral Drug Discovery (AViDD) U19 Center composed of an international consortium of scientists. Its views do not necessary reflect those of the National Institutes of Health.

## Data Availability

No data is associated with this article. Zenodo: ASAP Policy on Intellectual Property Management and Open Science Disclosure, https://doi.org/10.5281/zenodo.12191567 ^15^. Data are available under the terms of the Creative Commons Attribution 4.0 International license (CC-BY 4.0).
