# Supplementary material for: Enabling equitable and affordable access to novel therapeutics for pandemic preparedness and response via creative intellectual property agreements
Source: Wellcome Open Res. 2024 Jul 15;9:374. [Version 1] doi: 10.12688/wellcomeopenres.22645.1 (PMC11342024; doi:10.12688/wellcomeopenres.22645.1)
Supplement: Peer review history

# Reviewer report: Reviewer response for version 1

**DOI:** 10.21956/wellcomeopenres.24946.r91139

**Published:** 2024-09-20

**Authors:** Nat Moorman

**Affiliations:** 1 University of North Carolina, Chapel Hill, NC, USA

This is an exceptionally useful real-world account of the benefits and drawbacks of intellectual property protection in the context of global public health threats. The opinions and perspectives provided are well-reasoned and consider alternate views. While all may not agree with all points made, this article provides a useful firsthand perspective that should generate significant discussion and thought amongst those focused on antiviral drug development.

Does the article adequately reference differing views and opinions?

Yes

Are all factual statements correct, and are statements and arguments made adequately supported by citations?

Yes

Is the Open Letter written in accessible language?

Yes

Where applicable, are recommendations and next steps explained clearly for others to follow?

Yes

Is the rationale for the Open Letter provided in sufficient detail?

Yes

Reviewer Expertise:

Virololgy, antiviral drug discovery and development

I confirm that I have read this submission and believe that I have an appropriate level of expertise to confirm that it is of an acceptable scientific standard.

# Reviewer report: Reviewer response for version 1

**DOI:** 10.21956/wellcomeopenres.24946.r91140

**Published:** 2024-08-22

**Authors:** Paul Willis

**Affiliations:** 1 Medicines for Malaria Venture, Geneva, Geneva, Switzerland

The paper describes the IP experiences of the Open Source COVID moonshot project and the targeted IP strategy that was adopted to overcome challenges in the further development of the compound.

 This indexing makes a valuable and significant contribution to the Open Science/IP strategy debate.  While patents within the pharmaceutical industry are traditionally used to recoup investments, here targeted IP is proposed as a method to facilitate drug candidates being developed for affordable and equitable access.

 As outlined, to obtain IP protection on a candidate from an open-source project, at some point data release needs to be delayed.  Data already in the public domain could be cited as prior art, so it is critical that a team identifies the point where data/structure release is paused.  Projects often take time to identify that a compound/sub-series is a potential candidate/close to a candidate, so this ‘secrecy point’ could be challenging to spot: key assay data can take time to collect, and teams often feel future analogues will be even better.  It would therefore be helpful if, in future publications, the team can discuss their learnings on the timing of this decision.

 A particular approach to the development of compounds from open-source drug discovery projects is discussed.  Other open-source teams may choose to maintain a no-IP approach. The paper states that a ‘straight to generic’ drug discovery is appropriate in some domains.  In future presentations/papers the team could perhaps provide more details on how such examples can be identified.

 The paper states that ‘in commercial drug discovery, patents are generally as broad as possible, with the goal of excluding competitors'.  A potential disadvantage of broad patents is that they can be more difficult to defend/obtain a granted patent.  There is an alternative school of thought that narrow focused patents can be advantageous.

 The paper also makes insightful contributions to the debate around having molecules with clinical data available to allow a rapid response to any future pandemic.

Does the article adequately reference differing views and opinions?

Yes

Are all factual statements correct, and are statements and arguments made adequately supported by citations?

Yes

Is the Open Letter written in accessible language?

Yes

Where applicable, are recommendations and next steps explained clearly for others to follow?

Yes

Is the rationale for the Open Letter provided in sufficient detail?

Yes

Reviewer Expertise:

Drug discovery and development targeting affordable drugs with equitable access, also open source drug discovery

I confirm that I have read this submission and believe that I have an appropriate level of expertise to confirm that it is of an acceptable scientific standard.

# Reviewer report: Reviewer response for version 1

**DOI:** 10.21956/wellcomeopenres.24946.r91136

**Published:** 2024-08-16

**Authors:** Robert Jordan

**Affiliations:** 1 Bill and Melinda Gates Foundation, Seattle, Washington, USA

1. The authors of this manuscript describe a model for intellectual property protection designed to maximize equitable access to novel therapeutics for diseases with high public health need but low commercial value. This is an important concept essential for developing antivirals for pandemic preparedness.
2. For diseases with established commercial markets, pharmaceutical companies rely on patents to safeguard their intellectual property, thereby controlling manufacturing and distribution. This system enables companies to set prices based on market dynamics, ensuring a return on investment to cover discovery and development costs. Consequently, such products tend to be expensive and primarily accessible only in high-income countries.
3. Corporate philanthropy, through access programs, often falls short in meeting the needs of patients in low-income regions. While open access science could enhance the availability of novel therapeutics by facilitating the free exchange of information and intellectual property, the absence of intellectual property protection can deter private sector investment, which is crucial for bringing products to market.
4. To address this issue, the authors suggest a hybrid approach where patents are filed only to protect critical intellectual property, while early preclinical data is freely shared in open access journals. This model, described as ‘maximally permissive licenses’ based on ‘minimally defensive patents,’ aims to balance the need for global access with the incentives for innovation.
5. This approach enables the generation of a "minimum" therapeutic asset for global distribution without hindering innovation or the development of next-generation products that can be patented and commercialized for investment returns. The strategy is designed to ensure equitable access to first-generation therapeutics while allowing commercial developers to improve upon these discoveries and create better medicines for commercial sale.
6. The authors propose that patent owners negotiate licensing terms with commercial developers to maximize distribution and minimize delays. Exclusive licenses could incentivize commercial developers to manufacture and sell therapeutics in high-income countries, but it is crucial that these exclusive license agreements include equitable access terms to ensure fair distribution. In addition, provisions for sub-licenses for generic manufacturing activated during an outbreak would also increase the chances that drugs would be globally distributed. The authors should also address how these terms would be enforced.
7. A potential concern is that companies might use open access data to develop differentiated compounds, which could result in blocking patents and hinder the distribution of first-generation therapeutics. However, this scenario is deemed unlikely due to the public and political pressure that would likely encourage ethical practices during a pandemic. The authors should address this potential risk and explore possible mitigation strategies for their intellectual property protection model.
8. The authors acknowledge the commercial challenges of developing antivirals for pandemic preparedness. Their approach, which emphasizes minimal patent protection, benefits patients without stifling innovation and provides a framework for product development partnerships funded by government and non-governmental organizations. This ensures the availability of antiviral therapeutics in the event of an outbreak. In summary, this manuscript introduces a crucial concept often overlooked in the development of products for global populations, especially in therapeutic areas with limited market incentives. This important work offers a solution for developing therapeutics for diseases impacting global health and provides a framework for engaging the private sector in their development.

Does the article adequately reference differing views and opinions?

Yes

Are all factual statements correct, and are statements and arguments made adequately supported by citations?

Yes

Is the Open Letter written in accessible language?

Yes

Where applicable, are recommendations and next steps explained clearly for others to follow?

Yes

Is the rationale for the Open Letter provided in sufficient detail?

Yes

Reviewer Expertise:

Antiviral research

I confirm that I have read this submission and believe that I have an appropriate level of expertise to confirm that it is of an acceptable scientific standard.
